# Working in Aluminum

**Published:** 1861-09

**Authors:** 


### Selections.

Working in Aluminum.—We find the following valuable article in the *Ironmonger* (English.) The information was obtained from Messrs. Bell & Brothers, of Newcastle-on-Tyne, manufacturers of aluminum, by Professor Deville's process, and will be very useful to many of our readers.

The peculiar properties of this substance having been so little understood, has hitherto hindered its general employment, but now that it is sold in a pure state at as low a rate as 50s. per pound avoirdupois, it is likely to be much more frequently used.

Aluminum is a metal of fine white color, slightly inclining to blue, especially after being well hammered when cold.

Aluminum, like silver, is susceptible of a very fine "matting," which is not affected by exposure to the air, or by any of the impurities usually present in the atmosphere of towns. To obtain this matting, the aluminum objects (being previously washed in benzole or essence of turpentine) must be plunged into a weak solution of caustic soda, thoroughly well washed, and exposed to the action of strong nitric acid. When the desired matting has been obtained, it must be well washed again, and dried in sawdust.

Aluminum is easily polished or burnished. To do this, it is necessary to use a mixture of equal parts of rum and olive oil, as an intermediate substance between it and the polishing stone or powder used. The polishing stone, steeped in this mixture, will then burnish aluminum in the same manner as gold and silver is burnished, care being taken not to press too heavily upon the burnishing instrument.

Aluminum can be beaten out, either hot or cold, to the same extent and as perfectly as gold or silver; and it is susceptible of being rolled in much the same way as either of the above metals. Leaves as thin as those used for gilding and silvering can be made of aluminum. Covered ingot molds of iron answer best for receiving aluminum intended to be used in the rolling mill. Aluminum quickly loses its temper, and therefore requires frequent reheating. The temperature of this reheating is a dull red heat, and when the plates become very thin, this demands the greatest attention.

Aluminum is easily drawn into wire. For this, the ingots are run into an open mold, so as to form a kind of quadrangular shape of a little less than half-inch section, which is then beaten upon the edges by the hammer very regularly; the operation of drawing out is then commenced on a horizontal bench, by very gradually reducing the diameter of the metal intended to be drawn into wire, and, by frequent reheating, and then the ordinary process of wire-drawing can be proceeded with. When the threads are required extremely fine—as, for example, for the manufacture of lace—the heating becomes a very delicate operation, on account of the fineness of the threads and the fusibility of the metal. The heat of the current of air issuing from the top of the glass chimney of an Argand lamp will suffice for the heating.

The elasticity of aluminum is very much the same as that of silver, and its tenacity also about the same. The moment after it has been melted, aluminum possesses about the hardness of pure silver; when it is hammered out, it almost resembles that of soft iron; it becomes elastic, acquiring at the same time considerable rigidity, and emits the sound of steel when suffered to fall upon a hard body.

A property which aluminum manifests in a high degree is that of excessive sonorousness. This property has already rendered it of service in the construction of several musical instruments.

Aluminum is much lighter than ordinary metals. Its density is 2.56, a quarter that of silver, and about a third that of iron. By the action of the hammer, the density of aluminum increases sensibly, so as to become equal to 2.67.

Aluminum melts at a higher temperature than zinc, and a lower one than silver; to melt it, an ordinary earthenware crucible must be employed, without the addition of any sort of flux.

Its low point of fusion, along with its slowness of heating, require that for melting it a less intense heat should be used, but applied for a longer time than in melting silver.

It is easily melted in an open crucible, which facilitates the removal of the dust and other impurities which appear on the surface of the metal; and for the purpose of stirring the entire mass, a clean iron spatula is used.

Aluminum is easily run into metallic molds; and, still better, for objects of a complicated form, into molds of dry porous sand, formed so as to allow an easy passage for the air expelled by the metal, which is viscous when melted. It ought to contain a greater number of passage holes, and should be so managed as to run it in one long and perfectly cylindrical git. When heated to a red heat, it ought to be poured out with tolerable rapidity. A small portion of the fused metal should be caused to run into the git itself when full, to compensate for the contraction of the substance of the metal at the moment of solidification.

By following all these precautions, castings of the highest degree of fineness may be obtained; but, at the same time, to succeed perfectly, an especial acquaintance with the subject is needed.

In the production of work where the use of the lathe becomes necessary, any scratching or tearing of the metal by the tool is avoided by covering the surface to which the tool is applied with a varnish composed of stearic acid and essence of turpentine.

When aluminum is soiled by greasy matters, it can be cleaned by benzole; if it be soiled by dust only, india rubber or very weak soap and water may be used.

The pieces of aluminum intended to be soldered must be prepared in the same manner as objects are treated for soldering with tin, viz., by a "tinning;" but it must be remembered that it is indispensable that this tinning must take place with the solder itself. The pieces to be soldered, thus tinned beforehand, are afterward joined together and exposed to the flame either of a gas blowpipe, or any of the ordinary sources of heat used in such cases. In order to unite the solderings, small tools of aluminum are used. These tools are used as little soldering instruments, and they facilitate at the same time the fusion of the solder and its adhesion to the previously prepared aluminum.

The use of tools of copper or brass used when soldering gold and silver, must be strictly avoided, as they would form colored alloys with the aluminum and the solder. It is of the greatest importance never to use any flux to cause the solder to melt, as all those at present known attack aluminum, and prevent the adhesion of the pieces to be soldered. The use of the little tools of aluminum is an art which the workmen must acquire by practice ; in fact, at the moment of fusion the solderings must have the friction applied, as they melt suddenly in a complete manner. In soldering aluminum, it is well to have both hands free, and to use only the foot for the blowing apparatus.

Solders of different compositions and degrees of fusibility have been employed in soldering aluminum. The following are those which have been generally used, ranged according to their order of fusibility :—

| | 1. | 2. | 3. | 4. | 5. |
|---|---|---|---|---|---|
| Zinc | 80 | 85 | 88 | 90 | 94 |
| Copper | 8 | 6 | 5 | 4 | 2 |
| Aluminum | 12 | 9 | 7 | 6 | 4 |

No. 4 is the one usually preferred, particularly for soldering smaller objects.

In order to make the solder, the copper is first melted, the necessary aluminum is added, and stirred by means of an iron spatula, unpolished, as it comes from the blacksmith, adding also a little tallow ; the zinc is then added, avoiding too much heat, as this last metal is easily oxydized and is very volatile. —*Scientific American.*

